# Portal vein aneurysm, a pathological entity or an innocent bystander? A review of six cases

**DOI:** 10.1259/bjrcr.20220073

**Published:** 2022-11-01

**Authors:** Nada Majeed, Badr Bannan, Niaz Ahmad, Zergham Zia, Majed Ashour

**Affiliations:** 1 Departmet of Diagnostic and Interventional Radiology, King Faisal Specialist Hospital and Research Centre, Jeddah, Saudi Arabia; 2 Department of Surgery, Section of Transplantation and Hepatobiliary Surgery, King Faisal Specialist Hospital and Research Centre, Jeddah, Saudi Arabia

## Abstract

Portal venous aneurysms (PVAs) are a rare venous aneurysms. The mean diameter of a healthy portal vein varies considerably, with maximum diameter of 15 mm in healthy subjects and 19 mm in cirrhotic patients.

The presentation varies; they could come with abdominal pain or more often as an incident imaging finding. Although risk factors like portal hypertension and liver cirrhosis have been highlighted, the aetiology remains to be clarified. PVA may be associated with various complications: thrombosis, aneurysmal rupture, inferior vena cava obstruction or duodenal compression. A conservative treatment showed satisfying clinical and radiological response, however, surgical and endovascular options can be considered.

The aetiology and the mechanism of formation of PVA remain ill-defined. We aimed to use the small cohort of cases to define the distribution and radiological features of PVA and not for determining its prevalence or details of management.

We retrospectively reviewed six cases from our institution (King Faisal Specialist Hospital and Research Centre, Jeddah) with variable presentations, complications and outcomes.

Our review revealed that portal venous system aneurysms were mostly incidental, single, not gender- or age-specific and were frequently (66%) intrahepatic. Main portal vein was involved in three cases and splenic vein in only one case. Most of the portal venous system aneurysms were fusiform in configuration.

Although PVAs are rare, more cases are detected through imaging. Hepatobiliary surgeons, gastroenterologists and radiologists should be aware of this entity, as it can have a wide variety of clinical spectrum.

Our review and the limited evidence in published literature suggest that an individualised multidisciplinary team approach should be adopted to decide the best management and outcomes for each patient.

## Introduction

Portal venous aneurysms (PVAs) are the most common visceral venous aneurysms representing 3% of all venous aneurysms.^1^ The most common reported sites for their development are the main portal vein and the confluence of the splenic and the superior mesenteric veins.^2^ The patient may present with abdominal pain though more commonly PVA is an incidental finding. Whilst the risk factors like portal hypertension and liver cirrhosis have been highlighted, the aetiology remains unclear. PVA may lead to significant complications such as thrombosis, aneurysmal rupture, inferior vena cava obstruction and duodenal compression. A conservative management approach is satisfactory in most cases. An intervention may be considered for symptoms, increasing size of the PVA and PV thrombosis. In such cases, radiological intervention is the mainstay of management and surgery is reserved where radiological intervention is inappropriate or not successful.

## Case presentation

A summary of the cases in this cohort is presented in Table 1.

**Table 1.** Demographic, clinical and imaging characteristics of six patients with portal vein aneurysm

	Age	Sex	Indication for initial study	Lab works	Associated abnormality	Treatment	Site of involvement
1	21 y	F	Epigastric pain, 1 month	Protein C, & Protein S are negative. PNH is negative.antiphospholipid –is negative.	PV Thrombosis & cavernous transformation Portal hypertension GI bleeding	Anticoagulation Portal vein recanalisation	Main portal vein Intrahepatic portion of main portal vein
2	29 y	F	RUQ pain, 1 year, referred for liver lesion	-	Multiple hepatic FNH lesions	Conservative	Left intrahepatic portal vein
3	61 y	F	Recurrent periumbilical & right-sided abdominal pain	–	–	Conservative	Main portal vein
4	76 y	F	Hepatitis C cirrhosis, Portal hypertension with oesophageal varices and hepatic encephalopathy	–	Thrombosis	Conservative	Intrahepatic anterior branch of right portal vein
5	25 y	M	Living kidney donor CT Angiogram pre-donation	–	–	Conservative	Confluence of portal vein extending to left main portal vein
6	36 y	M	Living kidney donor CT Angiogram pre-donation	–	–	Conservative	Main portal vein—Splenic vein

GI, gastrointestinal.

### Patient 1

**Figure 1.**
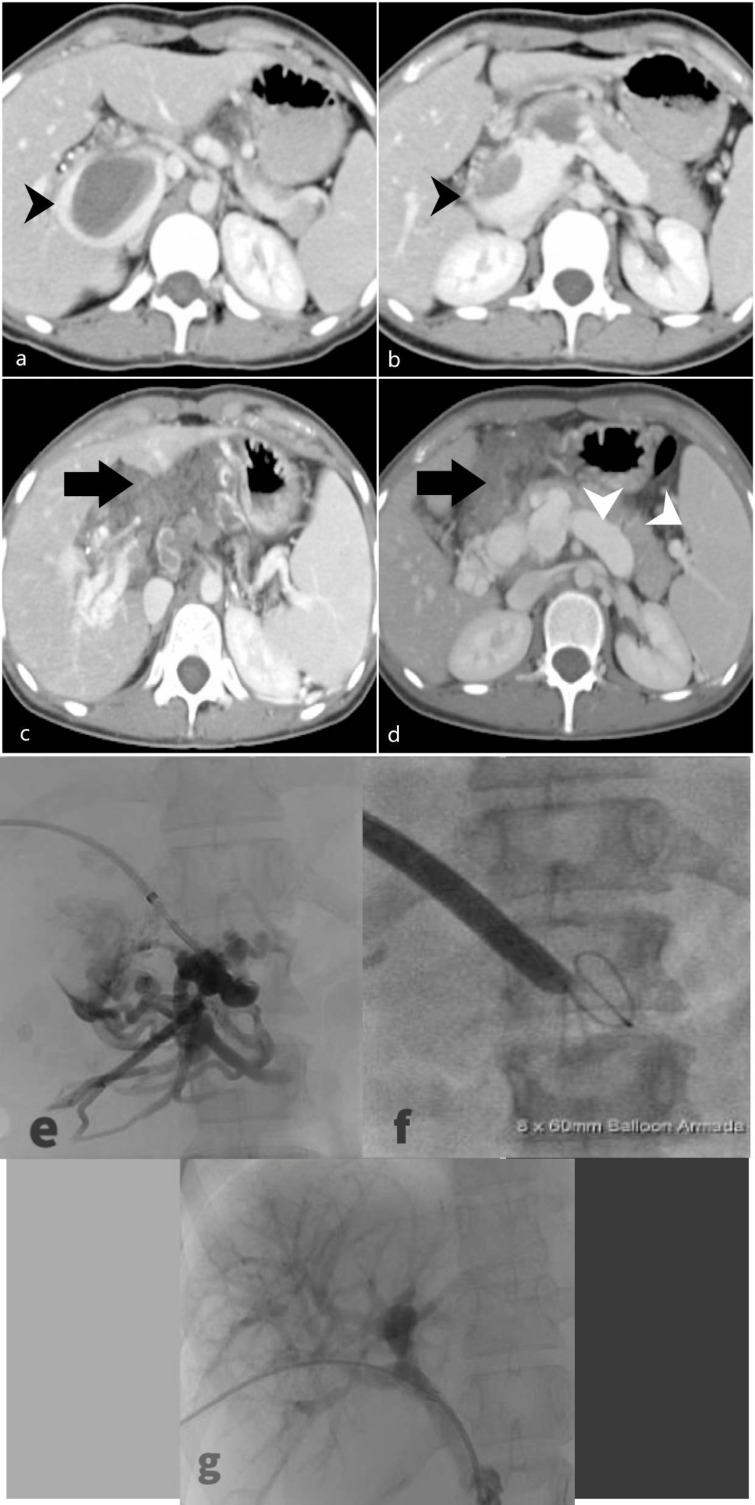
(**a, b**) Axial CT images in the portal venous phase showed a 4 cm aneurysmal dilatation of the intrahepatic portion of main portal vein. A large filling defect of varying densities seen within, which likely represent different ages of a thrombus (black arrowhead). (**c, d**) Follow-up CT scan 2 years later showed chronic thrombosis of the main portal vein with cavernous transformation (black arrow) with an apparent acute thrombus seen within the collateral vessels. The distal superior mesenteric vein is aneurysmal and splenic vein is dilated with progressive splenomegaly, related to portal hypertension (white arrowhead). (**e**) Portal venogram demonstrating occlusion of the extrahepatic main portal vein. (**f**) Portal vein access balloon dilatation of the vein was performed. (**g**) Post-venoplasty venogram showed adequate flow in the portal vein.

21-year-old female with no known comorbidity, presented with epigastric pain for 1 month. CT abdomen revealed a 4 cm aneurysmal dilatation of the main portal vein including the intrahepatic part, with intramural thrombosis (**Figure 1, a and b**). A detailed thrombophilia work-up was negative including paroxysmal nocturnal hemoglobinuria (PNH), anti- phospholipid syndrome, protein C and protein S were all within normal limits. She was not tested for JAK2 mutation. She was commenced on oral anticoagulation with warfarin. While on warfarin, the patient had two episodes of life-threatening upper gastrointestinal (GI) bleeding after which warfarin was stopped. Follow-up CT scan 2 years later demonstrated chronic thrombosis of the main portal vein with secondary cavernous transformation and evidence of portal hypertension (**Figure 1, c and d**). A successful portal vein recanalisation was performed and balloon angioplasty was performed (**Figure 1, e, f and g**) following which she was commenced on therapeutic anticoagulant, *i.e.* Clexane.

### Patient 2

**Figure 2.**
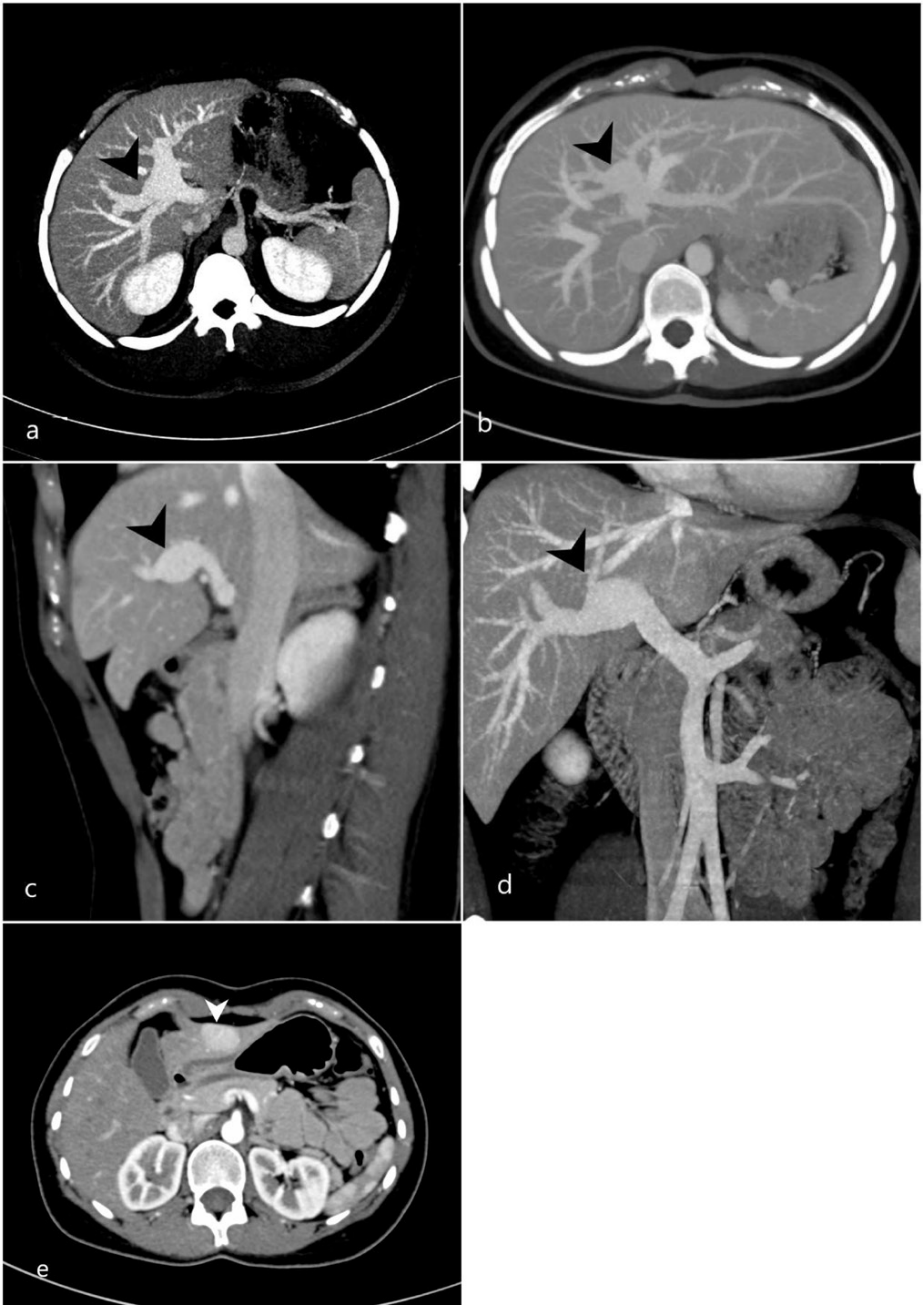
Axial (**a, b**), sagittal (**c**) and coronal (**d**) MIP images of an enhanced CT scan in portal venous phase showed a left intrahepatic portal vein aneurysm, measuring 1.6 cm in diameter (black arrowhead). (**e**) an incidental homogeneously enhancing lesion consistent with a focal nodular hyperplasia (white arrowhead). MIP, maximum intensity projection.

29-year-old female with no known comorbidity presented with a history of intermittent right upper quadrant abdominal pain for 1 year. CT abdomen demonstrated an incidental aneurysmal dilatation of the left intrahepatic portal vein, measuring 1.6 cm in diameter (**Figure 2, a, c, and d),** and an incidental single focal nodular hyperplasia (FNH) (Figure 2e). Follow-up MRI and ultrasound of the abdomen at 6 and 9 months respectively confirmed the stability of the liver lesion and of the PVA (not shown).

### Patient 3

**Figure 3.**
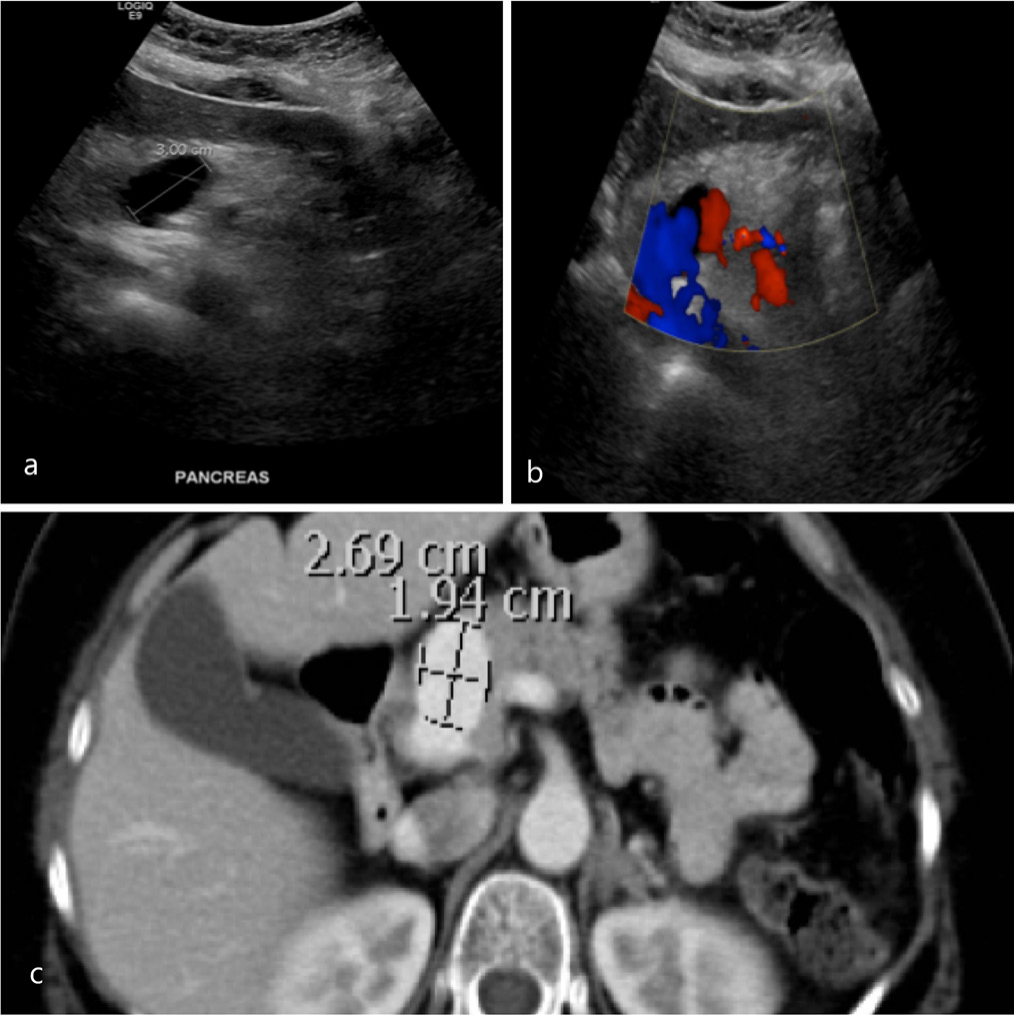
Greyscale (**a**) and colour Doppler ultrasound (**b**) of the abdomen demonstrated an anechoic cystic lesion measuring 3 cm near the head of the pancreas with an internal vascularity. (c) A contrast-enhanced CT scan in portal venous phase showed a main portal vein focal dilatation measuring 2.69 × 1.94 cm.

61-year-old female with a history of diabetes mellitus, hypertension, dyslipidaemia and a past history of stenting of bilateral carotid cavernous aneurysm presented with recurrent post-prandial periumbilical and right-sided abdominal pain. The pain was not associated with nausea or vomiting. history of pancreatitis. Ultrasound of the abdomen showed an anechoic cystic lesion measuring 3 cm near the head of the pancreas with internal flow (Figure 3, a and b). A contrast-enhanced CT scan of the abdomen demonstrated a 2.7 cm main portal venous aneurysm (Figure 3c). The patient was managed with symptomatic relief and regular surveillance of the aneurysm.

### Patient 4

**Figure 4.**
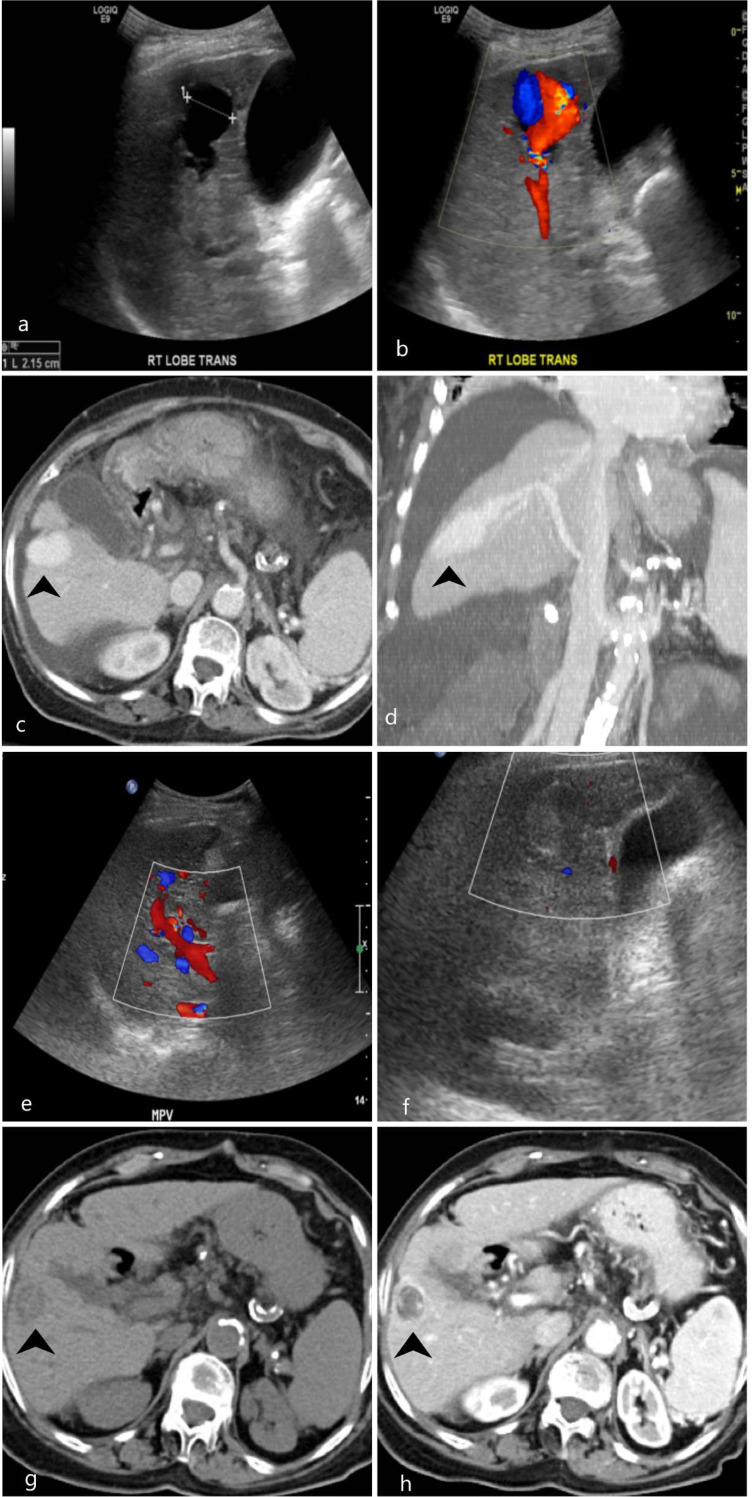
Greyscale (**a**) and colour Doppler ultrasound (**b**) of the abdomen demonstrated aneurysmal dilatation of the right intrahepatic portal vein with internal vascularity, measuring 2.15 cm in dimeter. An axial contrast-enhanced CT scan in portal venous phase (**c**) and a reformatted coronal MIP image (**d**) showed a focal aneurysmal dilatation of the intrahepatic anterior branch of the right portal vein measuring 2.3 × 2.5 cm (black arrowhead in c and d). Partial thrombosis of the right portal vein, extending into the main vein was also noted (white arrow in c). Cirrhotic liver and small ascites are noted. Follow-up greyscale (**e**) and colour Doppler (**f**) ultrasound scan after 4 years showed no colour flow within the right intrahepatic portal venous aneurysm, suggesting thrombosis, Non-enhanced CT scan (**g**) and enhanced CT abdomen in portal venous phase (**h**) confirmed thrombosis of the intrahepatic portal vein aneurysm (black arrowhead in g and h). Secondary cavernous transformation has also developed. MIP, maximum intensity projection.

76-year-old female had a history of hepatitis C cirrhosis, complicated by severe portal hypertension, oesophageal varices and hepatic encephalopathy. She had multiple episodes of life-threatening upper and lower GI bleed. A routine ultrasound (Figure 4, a and b) showed an aneurysmal dilatation of the right intrahepatic portal vein with internal vascularity, measuring 2.15 cm followed by an enhanced CT scan of the abdomen (Figure 4, c and d). that demonstrated cirrhotic features of the liver and an aneurysmal dilatation of the right intrahepatic portal vein measuring 2.5 cm in dimeter. Serial follow-up examination up to 4 years showed stability of the aneurysm. After 4 years, an ultrasound scan demonstrated thrombosis of the right intrahepatic portal venous aneurysm (Figure 4, e and f). This was subsequently confirmed by CT scan (Figure 4, g and h). The patient remains stable on the follow-up studies until the current date.

### Patient 5

**Figure 5.**
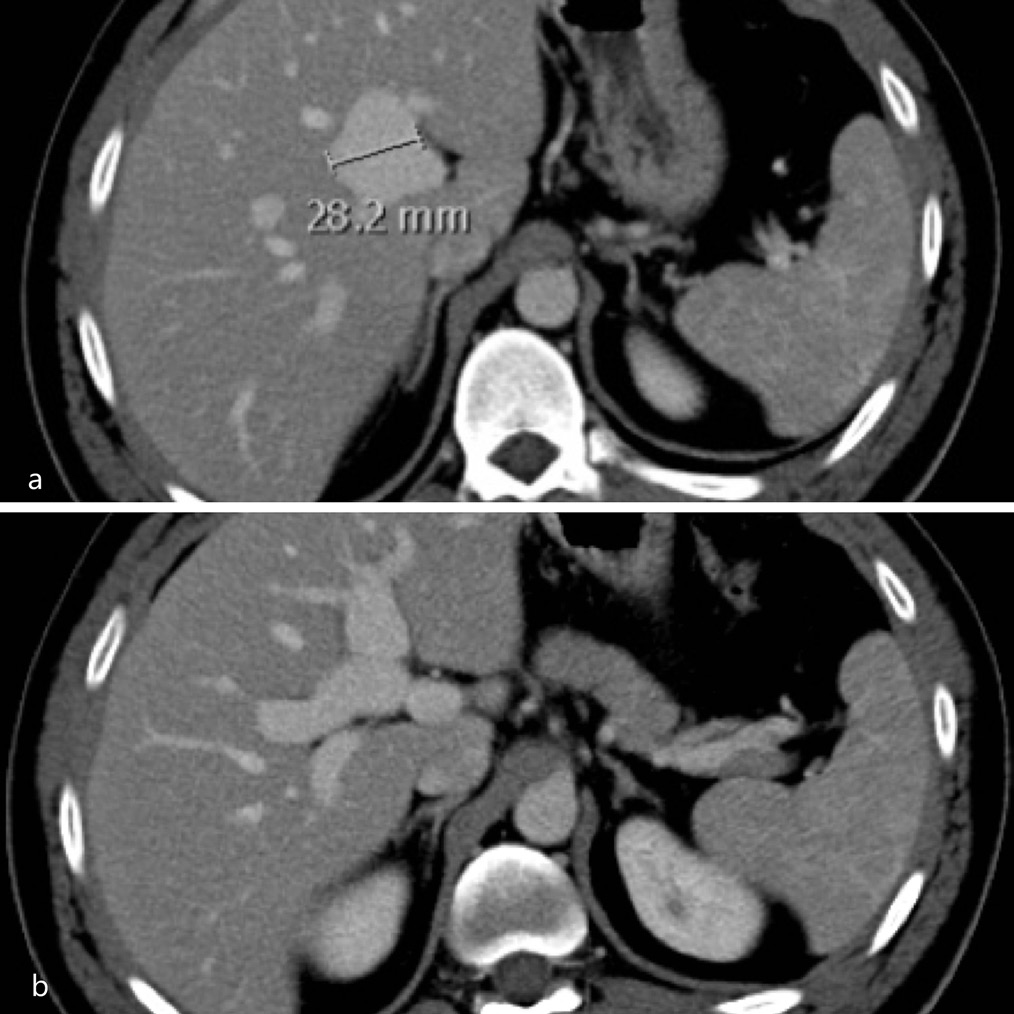
(**a, b**). Enhanced CT abdomen in portal venous phase demonstrated an aneurysmal dilatation involving the confluence of the portal vein extending into the left main portal vein. The maximum diameter at the confluence measures 2.8 cm.

A 25-year-old male with no known comorbidity underwent an abdominal CT scan as a part of living kidney donation work up. An incidental aneurysmal dilatation involving the confluence of the portal vein extending into the left main portal vein was demonstrated. The maximum diameter at the confluence measures 2.8 cm. The liver was unremarkable (**Figure 5, a and b**). The patient underwent an uneventful laparoscopic nephrectomy. The PVA was managed by annual ultrasound surveillance.

### Patient 6

**Figure 6.**
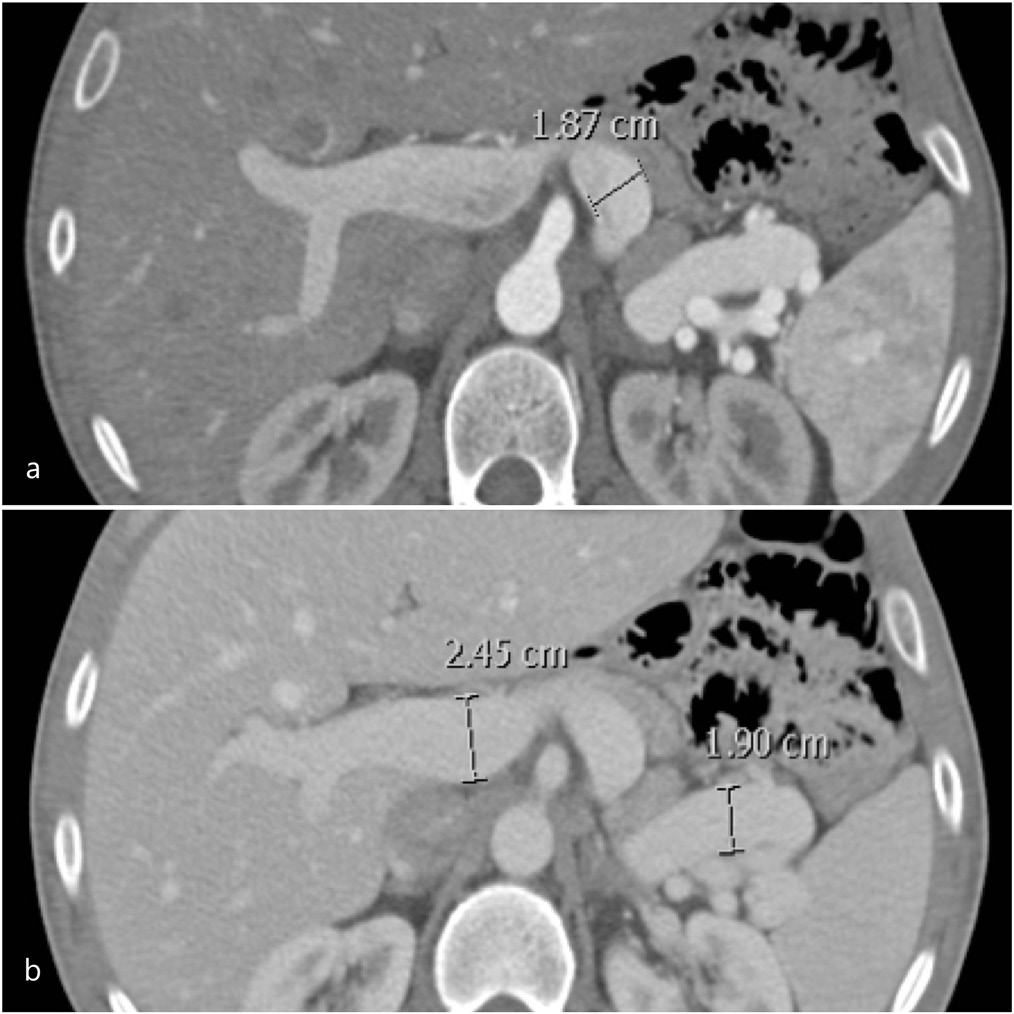
(**a, b**). Enhanced CT abdomen in portal venous phase showed a fusiform aneurysmal dilatation of the portal vein (2.45 cm) and splenic vein (1.87 cm). The splenic artery is also prominent (1.9 cm).

A 36-year-old male with no known comorbidity underwent an abdominal CT scan as a part of living kidney donor work up. An incidental fusiform aneurysmal dilatation of the portal vein (2.5 cm) and splenic vein (1.9 cm) was noted at the confluence. The splenic artery was noted to be prominent (**Figure 6,** a and b). The patient underwent an uneventful laparoscopic nephrectomy. The PVA was managed by annual ultrasound surveillance.

## Discussions

Visceral venous aneurysms are being increasingly reported partly because of the widespread availability and refinement of modern imaging techniques such as MR and CT imaging. The most common site for visceral venous aneurysms is portal system with almost 200 reported cases and a prevalence of 0.43%.^1^ Most frequent reported sites for PVA are the main portal vein and the splenic-superior mesenteric vein confluence.^2^

The mean diameter of a healthy portal vein varies considerably, with maximum diameter of 15 mm in healthy subjects and 19 mm in cirrhotic patients.^3^ A portal vein diameter of more than 20 mm is considered to be diagnostic for extrahepatic portal venous system aneurysm.^3^ The upper limit for the diameter of a portal vein for diagnosing an intrahepatic PVA is not yet determined. Published literature suggests that intrahepatic portal vein larger than 9 mm in diameter and significantly larger than the remaining segments of the same vein may be considered to be aneurysmal.^4^

The mechanisms and aetiologies of a PVA are not well understood. Portal hypertension and chronic liver disease have been identified as risk factors.^1^ Other causes such as pancreatitis, trauma and previous surgery have also been associated with PVA. A significant number of PVA cases do not present with any underlying liver disease.^2^ Neurofibromatosis 1, Marfan syndrome and hereditary haemorrhagic telangiectasia, which leads to weakness of vascular wall, are rare causes of venous aneurysm that may also be associated with PVA.^3^ The origin of PVA may also be associated with congenital defect of portal venous system. The failure of complete regression of the right vitelline vein may be responsible for a venous saccular enlargement, leading to aneurysm. In most of our cases, the patients did not present any of the risk factors described above, thus supporting a congenital cause.

The clinical presentation of a PVA is related to their size, the presence of complications (thrombosis, rupture) or of concomitant disease such as liver cirrhosis and portal hypertension. Small aneurysms often produce no symptoms. Patients may present with recurrent upper abdominal or epigastric pain; jaundice; or, rarely, GI bleeding. Portal hypertension may coexist or may be secondary to PVA thrombosis and may present as upper GI UGI) bleed and/or ascites.^1^ Rupture of a PVA is rare and presents with catastrophic abdominal bleeding. Large portal venous system aneurysms can cause compression of the duodenum or bile ducts, leading to obstructive jaundice and gastric outlet obstruction.^5^

Recurrent thrombosis can cause portal vein occlusion that results in acute or chronic symptoms of portal hypertension. In our study, two (33%) of the patients had portal vein thrombosis and were symptomatic. In patients with a thrombophilia defect, an aneurysm can trigger thrombus formation by causing turbulent flow and stasis. On the contrary, thrombosis of the portal vein may lead to the formation of a PVA.^1^

In our small cohort, PVA was associated with cirrhosis and portal hypertension in only one patient (16%). These findings support the hypothesis that chronic liver disease and portal hypertension may be contributory but are not essential factor in the development of portal venous system aneurysm.

The presence of multiple PVA have been reported in literature where the patients have been asymptomatic or had symptoms unrelated to the portal venous system. Symptomatic patients always exhibited aneurysms that were thrombosed, usually had larger aneurysms than those of asymptomatic patients, and often had multiple aneurysms.^1^ Our review revealed that portal venous system aneurysms were mostly incidental, single, not gender- or age-specific and were frequently (66%) intrahepatic. Main portal vein was involved in three cases and splenic vein in only one case. Most of the portal venous system aneurysms were fusiform in configuration.

Ultrasound scan is considered accurate and reliable modality to screen for PVA as it appears as an anechoic mass, with non-pulsatile, monophasic waveform on colour doppler. A thrombosed PVA may appear echogenic mimicking a solid mass. It is also useful for follow-up and monitor the growth. Ultrasound is also useful in differentiating a PVA from a hypervascular pancreatic mass, which can be seen with neuroendocrine tumours or metastatic disease, as seen in our third case.

Contrast-enhanced CT and MR are both useful in the setting of equivocal sonographic findings, in particular when differentiating slow flow from thrombosis. The use of venographic studies has been restricted to patients who require an interventional procedure.

The management of PVA largely depends on patient’s symptoms and management of other associated condition such as portal hypertension and portal vein thrombosis. No clear guidelines exist and each case should be managed in its own merit. In the setting of acute portal vein thrombosis, anticoagulation therapy is recommended resulting in complete or partial recanalisation in up to 80% of patients.^3^ Patients failing anticoagulation therapy, with extensive thrombus burden involving the splenic and superior mesenteric veins, or with symptoms related to aneurysm mass effect may be referred to percutaneous thrombolysis or thrombectomy.^2^ Portal vein recanalisation by radiological intervention may also be attempted in selected cases, as seen in our first case. If the portal vein aneurysm enlarges or recanalisation procedures fail, portosystemic bypass surgery or aneurysmorrhaphy can be performed. Portocaval, mesocaval, or splenorenal shunts decrease portal pressure and help prevent aneurysmal size progression.^6^ In the absence of portal hypertension, aneurysmorrhaphy is preferred as it restores portal vein laminar flow while preserving normal hepatic flow, and decreases stasis and resultant thrombosis.^7^

Some authors have considered clinically symptomatic patients and complete thrombosis of PVA as indications for surgery. Brock et al postulated that patients with thrombosis extending to splenic vein and superior mesenteric vein should undergo thrombectomy and restoration of portal vein anatomy; but complication rates of surgical management have not been reported.^7^ A conservative approach may have a lower complication rate as in Case 4 in our cohort.

Based on our small cohort, we would not consider the presence of symptoms or thrombosis as strict indications for surgery, and a conservative approach and follow-up in first instance even for a large aneurysm or extension to splenic vein-superior mesenteric vein is our approach. This approach is also supported by the low risk of aneurismal rupture (2.2%). In case of treatment failure, surgical treatment may be considered. In cases with portal hypertension and splenomegaly, the patient may benefit from splenectomy. It is hypothesised that splenomegaly results in increased flow within the portal vein, and therefore increased pressure leading to PVA. There are also other reports describing resultant regression of portal venous system aneurysm size following splenectomy.^8^

PVA in the transplanted liver is less frequently reported.^8^ In one reported case, the PVA arise from the donor portal vein and was discovered during routine surveillance. The patient developed portal vein thrombosis and splenomegaly, eventually progressing to collateral vessel formation, renal and hepatic failure, resulting in death.

We describe the radiological findings of PVA in our case series to define the distribution and radiologic features of PVA and not for determining its prevalence or details of management.

## Conclusion

PVA is a rare entity and is increasingly being diagnosed in both non-transplant and transplant setting. Whilst mostly asymptomatic and an incidental finding, occasionally PVA may be symptomatic and may lead to significant complications such as portal vein thrombosis & rupture of PVA. The aetiology and the mechanism of formation of PVA remain unclear. Our review and the limited evidence in published literature suggest that multidisciplinary and individualised approach is needed for each PVA case.

## Learning points

- Recognition of portal venous aneurysms and predisposing factors.
- Diagnosis, monitoring and surveillance imaging modalities.
- Multidisciplinary and individualised approach is needed for each PVA case.

## References

[1] Koc Z, Oguzkurt L, Ulusan S . Portal venous system aneurysms: imaging, clinical findings, and a possible new etiologic factor. AJR Am J Roentgenol 2007; 189: 1023–30. doi: 10.2214/AJR.07.2121 17954635

[2] Lee T-P, Lu HC, Chou Y-H, Tiu C-M, Chiou S-Y, Chiou H-J, et al . Portal vein aneurysm: A case report and review of the literature. Journal of Medical Ultrasound 2009; 17: 57–62. doi: 10.1016/S0929-6441(09)60016-3

[3] Labgaa I, Lachenal Y, Allemann P, Demartines N, Schäfer M . Giant extra-hepatic thrombosed portal vein aneurysm: A case report and review of the literature. World J Emerg Surg 2014; 9: 35. doi: 10.1186/1749-7922-9-35 24795777 PMC4008416

[4] Philips CA, Anand L, Kumar KNC . Symptomatic presentation of intrahepatic portal vein aneurysm. ACG Case Rep J 2014; 2: 14–15. doi: 10.14309/crj.2014.69 26157892 PMC4435349

[5] López-Machado E, Mallorquín-Jiménez F, Medina-Benítez A, Ruiz-Carazo E, Cubero-García M . Aneurysms of the portal venous system: ultrasonography and CT findings. Eur J Radiol 1998; 26: 210–14. doi: 10.1016/s0720-048x(96)01146-1 9518231

[6] Jin B, Sun Y, Li Y-Q, Zhao Y-G, Lai C-S, Feng X-S, et al . Extrahepatic portal vein aneurysm: two case reports of surgical intervention. World J Gastroenterol 2005; 11: 2206–9. doi: 10.3748/wjg.v11.i14.2206 15810096 PMC4305799

[7] Flis V, Matela J, Gadžijev E . Portal vein aneurysm: when to operate? EJVES Extra 2003; 5: 31–33. doi: 10.1016/S1533-3167(03)00025-6

[8] Schwope RB, Margolis DJ, Raman SS, Kadell BM . Portal vein aneurysms: A case series with literature review. J Radiol Case Rep 2010; 4: 28–38. doi: 10.3941/jrcr.v4i6.431 22470738 PMC3303410

